# Correct Patterning of the Primitive Streak Requires the Anterior Visceral Endoderm

**DOI:** 10.1371/journal.pone.0017620

**Published:** 2011-03-18

**Authors:** Daniel W. Stuckey, Aida Di Gregorio, Melanie Clements, Tristan A. Rodriguez

**Affiliations:** Molecular Embryology Group, Medical Research Council's Clinical Sciences Centre, Imperial College, London, United Kingdom; Instituto de Medicina Molecular, Portugal

## Abstract

Anterior-posterior axis specification in the mouse requires signalling from a specialised extra-embryonic tissue called the anterior visceral endoderm (AVE). AVE precursors are induced at the distal tip of the embryo and move to the prospective anterior. Embryological and genetic analysis has demonstrated that the AVE is required for anterior patterning and for correctly positioning the site of primitive streak formation by inhibiting Nodal activity. We have carried out a genetic ablation of the Hex-expressing cells of the AVE (Hex-AVE) by knocking the *Diphtheria* toxin subunit A into the *Hex* locus in an inducible manner. Using this model we have identified that, in addition to its requirement in the anterior of the embryo, the Hex-AVE sub-population has a novel role between 5.5 and 6.5dpc in patterning the primitive streak. Embryos lacking the Hex-AVE display delayed initiation of primitive streak formation and miss-patterning of the anterior primitive streak. We demonstrate that in the absence of the Hex-AVE the restriction of Bmp2 expression to the proximal visceral endoderm is also defective and expression of Wnt3 and Nodal is not correctly restricted to the posterior epiblast. These results, coupled with the observation that reducing Nodal signalling in Hex-AVE ablated embryos increases the frequency of phenotypes observed, suggests that these primitive streak patterning defects are due to defective Nodal signalling. Together, our experiments demonstrate that the AVE is not only required for anterior patterning, but also that specific sub-populations of this tissue are required to pattern the posterior of the embryo.

## Introduction

In mouse, the first definitive axis of the embryo to form is the anterior-posterior (A-P) axis. At approximately 5.25dpc, a group of visceral endoderm cells at the distal tip of the egg cylinder differentiate into a morphologically distinct tissue, known as the distal visceral endoderm (DVE). DVE cells adopt a tall, columnar epithelial morphology, distinguishing them from the surrounding squamous visceral endoderm [1], [2], [3], [4]. Shortly after its formation, the DVE tilts and begins to move unilaterally over the underlying epiblast [2], [4], [5]. The direction of this movement determines the prospective anterior of the embryo and the DVE, now referred to as the anterior visceral endoderm (AVE), is essential for correctly positioning the A-P axis (reviewed in [3], [6]).

Embryological and genetic analysis has demonstrated that the AVE is required for anterior patterning (reviewed in [7], [8], [9]). Microsurgical ablation of the AVE at the onset of gastrulation leads to forebrain truncations [10] and ablation at 5.5dpc abolishes the expression of anterior neuroectoderm markers [11]. Analysis of mouse mutants where gene function has been specifically lost in extra-embryonic tissues has further demonstrated the role of the AVE in forebrain specification [12], [13], [14].

The AVE has also been shown to inhibit primitive streak formation. Ectopic AVE transplantation experiments have indicated that the AVE represses posterior markers [1], [15] and analysis of mutants with defective AVE movements [1], [14], [16], [17], [18], or no AVE formation [19], [20], [21], have suggested that the AVE inhibits primitive streak formation in the anterior of the embryo. Analysis of *Cerl^−/−^;Lefty1^−/−^* compound mutants has shown that the AVE does this via the inhibition of Nodal signalling [22].

The AVE is thought to comprise multiple populations of cells expressing different molecular markers [4], [23]. The homeobox gene *Hex* is one of the earliest markers of the AVE [5] and the *Hex*-expressing cells of the AVE have been proposed to represent a different population of cells from those expressing *Lefty1* or *Cerl1* [23]. To date experiments carried out to address the role of the AVE have analysed the function of this tissue as a whole or the role of specific genes within the AVE but not the importance of specific sub-populations of AVE cells. To address what roles the Hex-AVE may have during A-P axis development we have knocked a Cre inducible diphtheria toxin A cassette (DTA) [24], [25] into the *Hex* locus. We demonstrate that in contrast to the reported roles of the AVE in repressing Nodal activity, *Hex*-expressing cells of the AVE are required at 6.5dpc to pattern the anterior primitive streak.

## Methods

### Mouse strains

**Figure 1:**
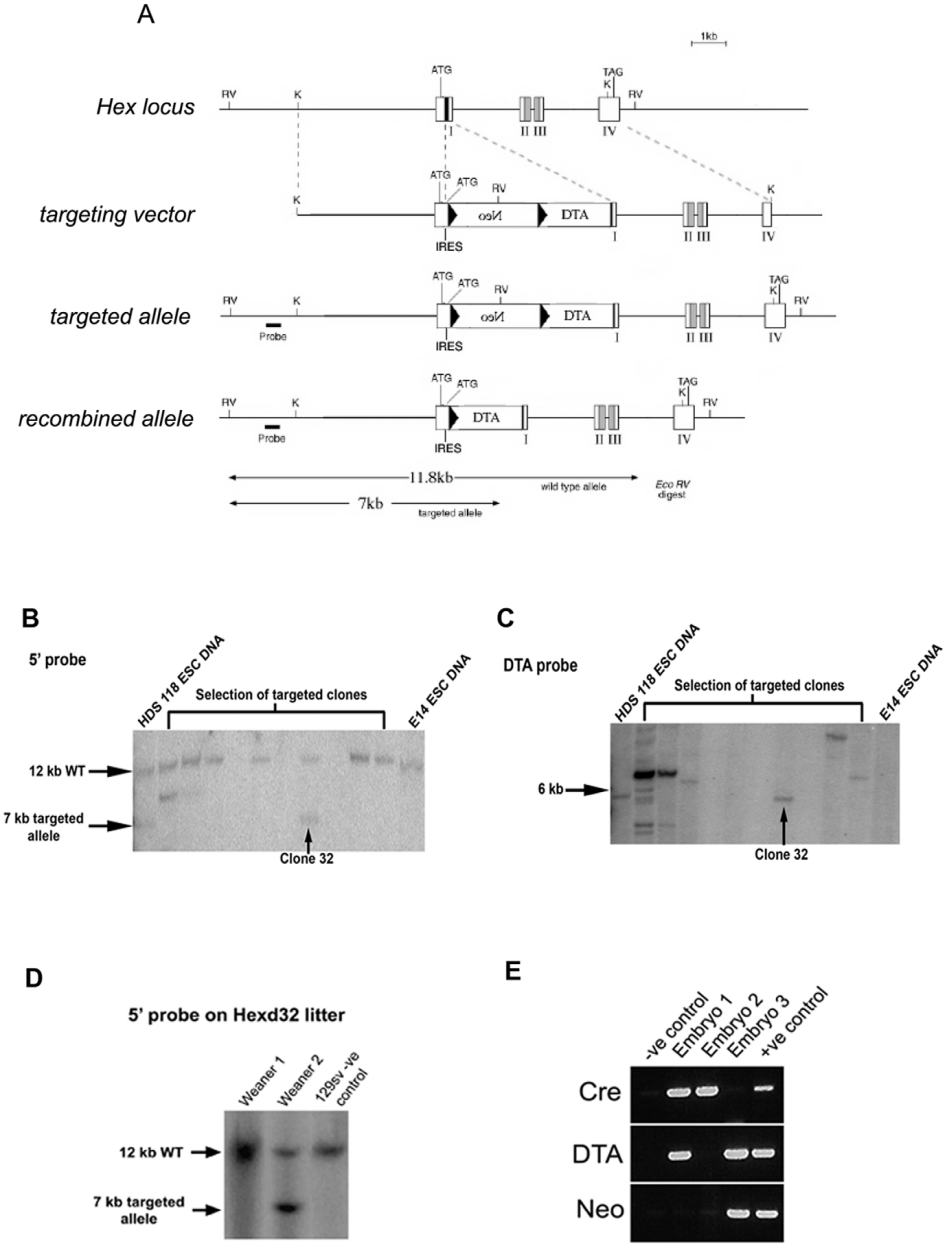
Genetic ablation of the AVE. (A) Strategy for knock-in of DTA into the *Hex* locus. (B) Identification of Hexd^+/−^ ES clones using an external 5′ probe. (C) Identification of Hexd^+/−^ ES clones using an internal DTA probe. (D) Identification of Hexd^+/−^ mice using an external 5′ probe. (E) Identification of Hexd^act/+^ embryos by PCR.

The *LoxPneoSTOP^R^LoxP-DTA* construct that has previously been used to ablate the roofplate [26], was introduced by gene targeting in ES cells into the *Hex* locus (Fig.1). Correctly targeted cells were used to generate Hexd mice and these were crossed to either β-actin*-Cre* [27] or *Sox2Cre* mice [28]. A small number of embryos resulting from the β-actin*-Cre* × Hexd cross escaped genetic ablation, were born, fertile and termed Hexd^act/+^. Genetic interaction experiments were performed using *Nodal-LacZ* mice [29]. *Hex-*GFP mice were used as a method of visualising *Hex*-expressing cells [30]. All mice were maintained and treated under the Home Office's animals (scientific procedures) Act 1986 under the Home Office approved Project Licence 70/5267.

### Genotyping of mice and embryos

DNA for genotyping was prepared according to standard procedures [31], [32]. Mice and embryos from the β-actin*-Cre* × Hexd cross were genotyped using the following primers DTA: CGACAATAAATACGACGCTGCGGG and CATCGCATCTTGGCCACGTTTTCC; Cre: CCAGCTAAACATGCTTCATC and CGCTCGACCAGTTTAGTTAC; neomycin: CAAGATGGATTGCACGCAGG and CGGCAGGAGCAGGTGAGAT; from the Hexd^act/+^ x Hexd^act/+^ cross using the neomycin primers and primers for the wild-type allele CGGAGGCGAATCTGAAGCCAG and GCATACAGCGGGACTCCCACG; *Hex*GFP mice using TGCAGTGCTTCAGCCGCTAC and CCAGCAGGACCATGTGATCG; and Nodal.Lac*Z* mice using CGCCAGCTGGCGTAATAGCGAAG and GATGGGCGCATCGTAACCGTGCA.

### Embryo culture

Embryos were cultured in 50 ng/ml BMP2 (R&D Systems) in pre-equilibrated drops. Each 80 µL drop comprised inhibitor diluted in DMEM:rat serum (1:1) covered with mineral oil. Embryos were incubated at 37°C, 5% CO_2_ overnight (typically 16 hours), then fixed in 4% paraformalde'hyde overnight at 4°C or stained for β-galactosidase activity.

### Whole-mount *in situ* hybridization (WISH) β-galactosidase staining and confocal acquisition

WISH was carried out as previously described [5]. Staining for Lac*Z* was carried out according to standard procedures [31]. For confocal analysis, embryos were stained with DAPI-Vectashield and TRITC-phalloidin mounting medium for 20 mins, mounted in 1∶1 Glycerol:PBS and analysed with a Leica DM IRB upright confocal microscope.

## Results

### Genetic ablation of the *Hex*-AVE between 5.5dpc and 6.5dpc

To genetically ablate the Hex-AVE we expressed diphtheria toxin subunit A (DTA) in the *Hex*-expressing cells of the AVE. This approach has been successfully used to ablate other cell populations within the embryo [26], [33]. We targeted the *LoxPneoSTOP^R^LoxP-DTA* construct, containing a Cre-inducible DTA, into the *Hex* locus to generate Hexd mice (Fig.1). Hexd mice were crossed to β-actin *Cre* mice to generate embryos with a DTA-expressing *Hex* allele, termed Hexd^act/+^. Analysis of this cross revealed that at weaning there were 52% fewer than expected Hexd^act/+^ mice. Given that close to mendelian numbers were observed at 9.5dpc, this data suggests that about half of the Hexd^act/+^ embryos died between this stage and weaning (Table S1). Those mutant animals that survived till weaning developed into apparently normal adults suggesting that the *Hex* locus carrying the DTA allele may be silenced during development in a proportion of embryos. When Hexd^act/+^ adult mice were crossed to wildtype mice we observed 53% fewer than expected mutant mice at weaning (Table S2), indicating the ablation system could be reactivated in the gametes of Hexd^act/+^ mice.

**Figure 2:**
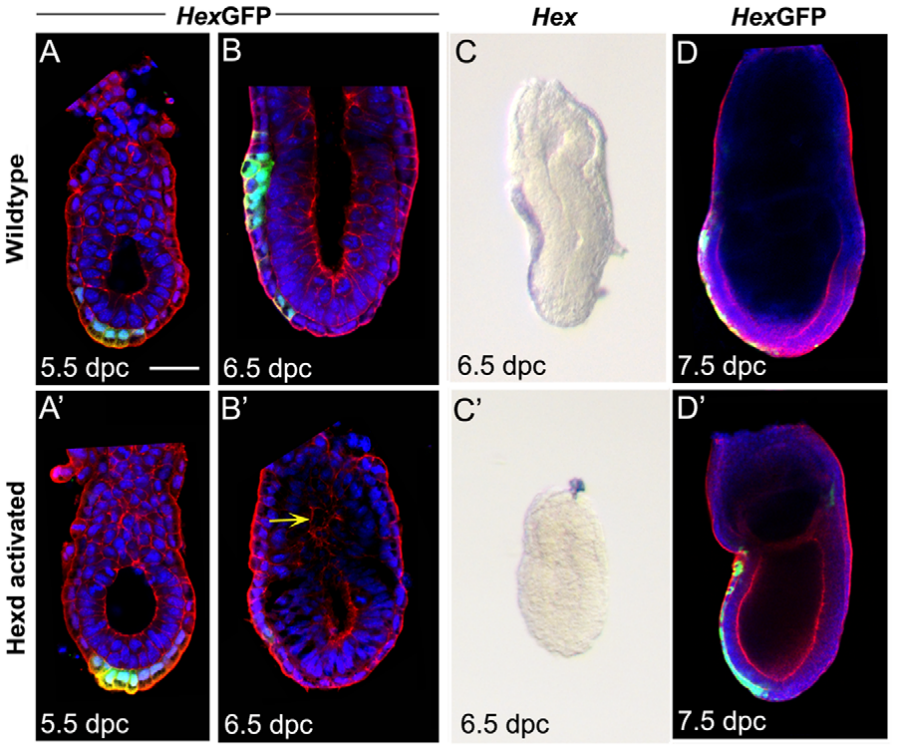
Ablation of the *Hex*-AVE in Hexd^act/+^ embryos. (A–D) *Hex-*GFP and *Hex* expression in wild-type and Hexd^act/+^ embryos. (A–A′) At 5.5dpc n = 1/7 Hexd^act/+^ embryos have lost expression. Green arrow points to *Hex-*GFP domain of AVE; (B–B′) at 6.5dpc n = 4/12 have severely reduced or lost expression, yellow arrow indicating disorganised pro-amniotic cavity; (C–C′) *Hex* transcript is reduced or lost in n = 6/6 Hexd^act/+^ embryos at 6.5dpc, but (D) not affected at 7.5dpc in these embryos, n = 0/4. Scale bar 40 µm in A–A″; 60 µm in B; 90 µm in C and 100 µm in D. GFP (green) Nuclear stain (blue); F-actin (red).

The *Hex*-GFP reporter line [30] provides a marker of *Hex* expressing cells that is independent of the *Hex* locus. We therefore analysed Hexd^act/+^ embryos carrying this reporter to address whether the *Hex* expressing cells were being ablated. Analysis of *Hex*-GFP expression at 5.5dpc revealed that the vast majority of Hexd^act/+^ embryos showed expression at this stage (n = 14/15; Fig.2A′). In contrast to this, at 6.5dpc 33% (n = 4/12) of Hexd^act/+^ embryos had lost *Hex*-GFP expression and presented a disrupted AVE (Fig.2B). These embryos also failed to show *Hex* transcript expression at this stage (Fig.2C). This data indicates that the Hex expressing cells of the AVE (Hex-AVE) had been ablated by 6.5dpc in one third of Hexd^act/+^ embryos. At 7.5dpc the vast majority of Hexd^act/+^ embryos were morphologically normal and all those analysed showed *Hex*-GFP expression in the definitive endoderm (Fig.2D) suggesting that those embryos with an ablated *Hex*-AVE were able to initiate gastrulation.

A very small proportion of Hexd^act/+^ embryos (4.4%; n = 11/250) at 6.5dpc and 7.5dpc were retarded and displayed a small, disorganised egg cylinder. Marker analysis on these embryos is presented in Fig.S1 but for the sake of clarity these embryos are not considered any further in the text.

### Ablation of *Hex*-expressing cells is accompanied by a reduction in other AVE markers

**Figure 3:**
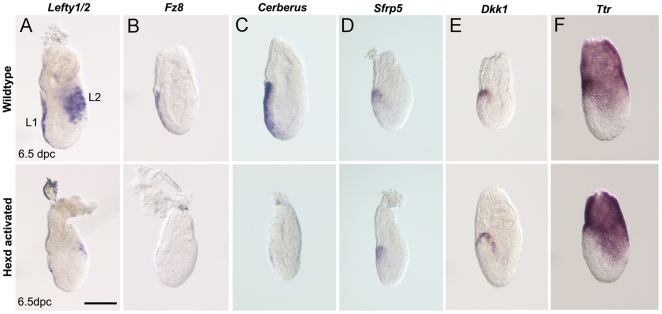
AVE defects in Hexd^act/+^ embryos. (A) *Lefty1/2*, (B) *Fz8,* (C) *Cerl,* (D) *Sfrp5,* (E) *Dkk1* and (F) *Ttr* expression in control and Hexd^act/+^ embryos at 6.5dpc. Hexd^act/+^ embryos with reduced or lost expression in the AVE =  *Lefty1/2* n = 13/28; *Fz8* n = 4/13; *Cerl*, n = 7/16; *Sfrp5*, n = 2/14; *Dkk1*, n = 0/15; *Ttr* normal in n = 16/16; *Lefty1/2 (L1 and L2)* lost from the posterior epiblast in n = 7/28. Scale bar 90 µm.

To further characterise the extent to which the AVE is affected in Hexd^act/+^ mutants, the expression of five AVE markers was analysed at 6.5dpc. WISH analysis revealed AVE patterning defects in 30% of Hexd^act/+^ embryos (n = 26/86), a similar proportion to the 33% of Hexd^act/+^ embryos that had lost *Hex*-GFP expression at 6.5dpc. These AVE patterning defects consisted of a vast reduction or total absence of *Lefty1/2*, *Fz8* and *Cerl1* transcript (Fig.3A–C) but little or no change in the expression of *Sfrp5* and *Dkk1*-two markers of the anterior portion of the AVE (Fig.3D–E). Despite the AVE patterning defects caused by ablation of the *Hex* expressing cells, the rest of the visceral endoderm remained intact and showed no evidence of a loss of integrity, as confirmed by the normal expression of *Ttr* at 6.5dpc (Fig.3F). These results suggest that between 5.5dpc and 6.5dpc the *Hex* domain of expression in the AVE (Hex-AVE) significantly overlaps with that of *Lefty1*, *Fz8* and *Cerl* and therefore is likely to mark a common population of cells. However, we cannot exclude that the loss of expression of *Lefty1*, *Fz8* and *Cerl* in Hexd^act/+^ embryos is due to a non-cell autonomous effect caused by the loss of the *Hex*-AVE. The lack of overlap with the *Sfrp5* and *Dkk1* domains suggests that these genes mark a different subpopulation of AVE cells from the *Hex* expressing sub-population at these stages. Furthermore, the fact that expression of these genes is correctly positioned at the anterior of Hexd^act/+^ embryos indicates that correct placement of cells expressing these genes is independent of the *Hex*-AVE after 5.5dpc.

### The patterning of the primitive streak is impaired after *Hex*-AVE ablation

**Figure 4:**
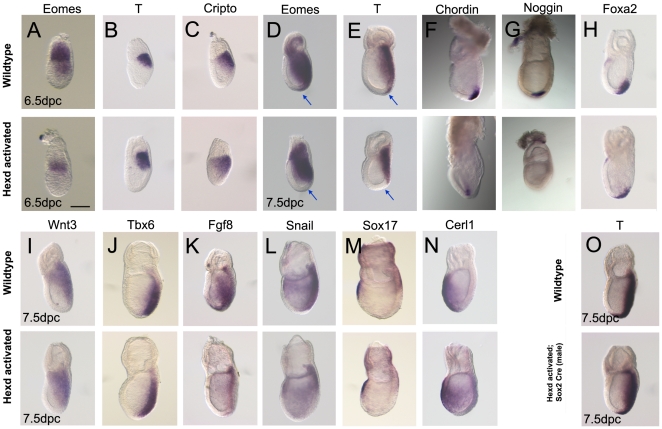
Defective primitive streak formation in Hexd^act/+^ mutants. (A–C) *Eomes, T* and *Cripto* expression in wildtype and Hexd^act/+^ mutant embryos at 6.5dpc. (D–N) Expression of *Eomes*, *T*, *Chordin*, *Noggin*, *Foxa2*, *Wnt3*, *Tbx6*, *Fgf8*, *Snail*, *Sox17* and *Cerl* in control and Hexd^act/+^ mutant embryos at 7.5dpc. Hexd^act/+^ embryos with normal expression: *T* (6.5dpc) n = 7/7; *Cripto* n = 9/9 *Wnt3* n = 11/11; *Tbx6* n = 13/13; *Foxa2* n = 8/8; *Cerl* n = 14/14; *Sox17* n = 14/14. Hexd^act/+^ embryos with reduced or lost expression *Eomes* (6.5dpc) n = 2/11; *Eomes* (7.5dpc) n = 3/18; *T* (7.5dpc) n = 3/14; *Chordin* n = 2/11; *Noggin* n = 6/15, *Fgf8* n = 5/17; *Snail* n = 3/14. (O) *T* expression in control and (O′) Hexd^+/−^;Sox2Cre^+/−^ embryos at 7.5dpc (n = 16/16). Blue arrow indicates the anterior primitive streak in D and E. Scale bar 90 µm.

To explore the consequences of *Hex*-AVE ablation, the expression of primitive streak markers were analysed in Hexd^act/+^ embryos. At 6.5dpc *Lefty2* and *Eomes* are normally expressed in the posterior epiblast, but the expression of both these genes were significantly reduced in Hexd^act/+^ embryos (Fig.3A and Fig.4A). In contrast, the expression of *T* and *Cripto* in the posterior epiblast was normal in Hexd^act/+^ embryos at this stage (Fig.4B–C). Together these data suggest that the onset of expression of some primitive streak markers is delayed when the AVE is ablated between 5.5-6.5dpc.

During gastrulation epiblast cells traverse through the primitive streak and adopt a mesodermal or endodermal fate (reviewed in [6]). WISH analysis revealed that whilst Hexd^act/+^ embryos displayed robust expression of *T* and *Eomes* in the extraembryonic mesoderm and intermediate primitive streak, these mutants displayed a lack of expression in the mesodermal derivatives of the anterior primitive streak (Fig.4D–E). In addition to these patterning defects, Hexd^act/+^ mutant embryos also exhibited reduced expression of both *Fgf8* and *Snail* in the anterior portion of the primitive streak (Fig.4K–L). We also observed a decrease or loss of expression of the anterior primitive streak markers *Chordin* and *Noggin* at 7.5dpc (Fig.4F–G), further suggesting that this region is affected in Hexd^act/+^ embryos. However, *Foxa2* was expressed normally in these embryos, indicating that not all anterior primitive streak markers/derivatives are affected (Fig.4H). Normal expression of *Wnt3* and *Tbx6* suggested that posterior and intermediate primitive streak formed normally (Fig.4I–J). These results indicated that some mesoderm cells were unable to extend anteriorly or were incorrectly patterned in AVE ablated embryos. In accordance with these subtle defects a third of Hexd^act^ embryos showed mild forebrain patterning defects at 9.5dpc (Fig.S2 and Table S3).

The observation that mesoderm markers are appropriately restricted to the posterior of the embryo indicates that the Hex-AVE is establishing the A–P axis in these embryos prior to its ablation. In total 24% (n = 24/100) of Hexd^act/+^ embryos showed defects in *T*, *Eomes*, *Chrodin*, *Noggin*, *Fgf8* or *Snail* expression between 6.5–7.5dpc, a similar proportion to the 30% of Hexd^act/+^ embryos with a miss-patterned AVE at 6.5dpc. Therefore it is likely that the anterior primitive streak patterning defects were arising due to ablation of the *Hex*-AVE.

To rule out the possibility that patterning defects in the anterior primitive streak were not simply due to ablation of *Hex*-expressing cells of the anterior definitive endoderm (ADE), we analysed the expression of the definitive endoderm markers *Sox17* and *Cerl*. The expression of neither of these markers was affected at 7.5dpc in Hexd^act/+^ embryos (Fig.4M–N). This, together with the normal *Hex*-GFP expression domain found in these embryos at this stage (Fig.2D), suggests that the ADE is not affected in mutant embryos. To confirm this observation, Hexd mice were crossed to male *Sox2*Cre mice, which activate DTA only in epiblast derivatives from 4.5dpc [28]. Unlike the observations in Hexd^act/+^ embryos, Hexd^+/−^;Sox2Cre^+/−^ embryos showed no change in the expression of *T* at 7.5dpc (Fig.4O) indicating that the primitive streak defects of ablated mutants were a consequence of *Hex*-AVE ablation.

### Ablation of the *Hex*-AVE leads to aberrant *Noda*l expression

**Figure 5:**
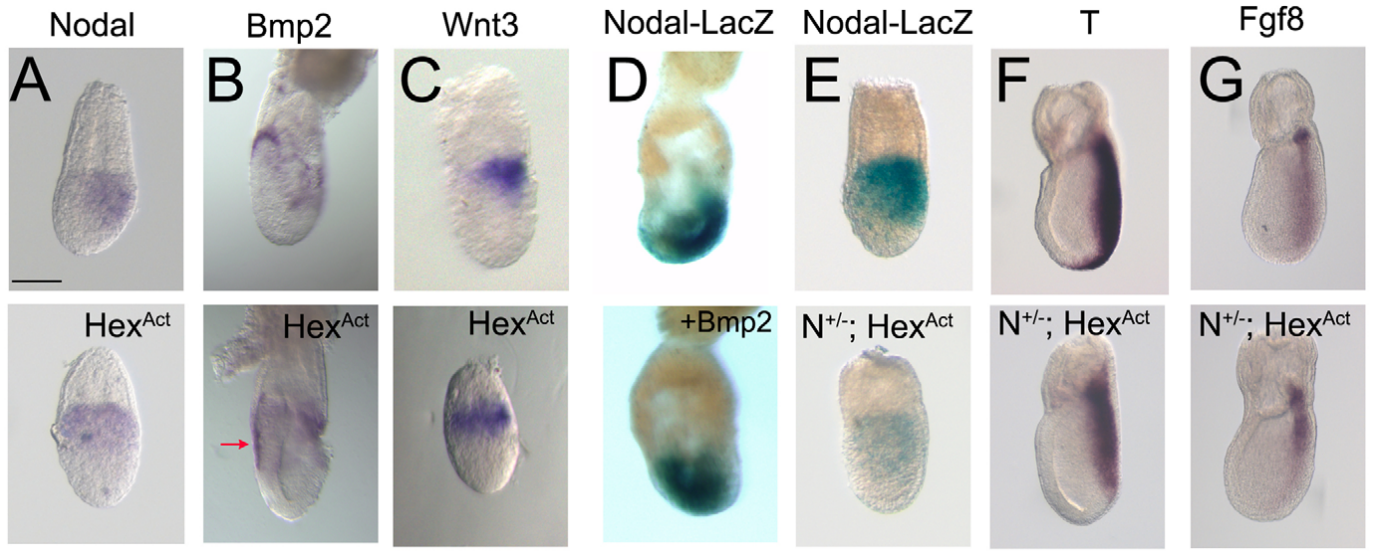
Nodal signalling is affected in Hexd^act/+^ embryos. (A) *Nodal* expression is not restricted to the posterior in Hexd^act/+^ embryos at 6.5dpc (n = 8/28). (B) *Bmp2* expression is expanded in the anterior visceral endoderm (red arrow) of Hexd^act/+^ embryos; n = 7/15. (C) Proximal expression of *Wnt3* in Hexd^act/+^ embryos; n = 3/6. (D) Culture with BMP2 leads to ectopic Nodal^LacZ/+^ expression (controls that are posterior n = 10/10; BMP2 treated posterior n = 1/8). (E) β-galactosidase expression in Nodal^LacZ/+^and Hexd^act/+^;Nodal^LacZ/+^ embryos at 6.5dpc (weak and proximal expression n = 6/8). (F–G) *T* and *Fgf8* expression in control and Hexd^act/+^;Nodal^LacZ/+^ embryos at 7.5dpc. Hexd^act/+^ embryos with reduced expression *T* n = 7/17 and *Fgf8* n = 6/11. Scale bar 90 µm.

The AVE and DVE secrete antagonists of Nodal signalling which restrict its activity to the posterior epiblast where it can induce the primitive streak (reviewed in [6]). However, Nodal signalling has also been implicated in the regulation of *Eomes* expression [34], which is defective in Hexd^act/+^ embryos. To test the effects of Hex-AVE ablation on Nodal signalling we analysed *Nodal* expression in Hexd^act/+^ embryos. At 6.5dpc, *Nodal* transcript is restricted to the posterior epiblast, but in 29% of the mutants *Nodal* expression was downregulated and found proximally with no posterior restriction (Fig.5A). This suggested that when the AVE is ablated between 5.5–6.5dpc, *Nodal* expression is not restricted to the posterior epiblast.

BMP4 has been shown to act at 6.5dpc in a positive regulation loop that amplifies Nodal signalling in the posterior epiblast via the activation of *Wnt3* [35]. We have also shown that BMP signalling is required to sustain *Nodal* expression throughout the epiblast prior to gastrulation [36]. *Bmp2* is expressed in the embryonic visceral endoderm at 5.0dpc [37] and in the AVE and posterior VE at 5.75dpc (Fig.S3), but at 6.5dpc is only observed in AVE cells that are adjacent to the embryonic-extraembryonic boundary (Fig.5B). Therefore *Bmp2* expression is extinguished from the AVE as *Nodal* expression is restricted to the posterior, suggesting it may be cooperating with BMP4 in maintaining *Nodal* expression prior to 6.5dpc [36]. Analysis of *Bmp2* expression in Hexd^act/+^ embryos shows that at 6.5dpc Bmp2 is expressed in all the anterior visceral endoderm and not just restricted to the anterior-most AVE as is seen in controls (Fig.5B and Fig.S3). Concomitant to *Bmp2* miss-expression, we observe that *Wnt3* expression, which is normally restricted to the posterior at this stage, is expressed throughout the proximal epiblast of Hexd^act/+^ embryos (Fig.5C). This indicates that the expression of a key element of the *Nodal* amplification loop is miss-localised at 6.5dpc.

To test whether BMP2 can sustain *Nodal* expression in a similar way to BMP4, we cultured 6.5dpc Nodal^LacZ/+^ embryos overnight in the presence of BMP2 recombinant protein. *Nodal-LacZ* expression in these embryos was found throughout the epiblast, and not restricted to the posterior as occurs in controls (Fig.5D). This suggests that BMP2 can maintain *Nodal* expression in the anterior epiblast. Therefore the expression of *Bmp2* throughout the anterior visceral endoderm at 6.5dpc in the *Hex*-AVE ablated embryos is one possible explanation for the miss-localised *Nodal* expression we observe in these embryos.

Hexd^act/+^ embryos display miss-patterning of the anterior primitive streak and these phenotypes are characteristic of decreased Nodal signalling [34], [38]. This, and the observation that we see miss-localisation of *Nodal* and *Wnt3,* led us to further investigate how Nodal signalling is affected in Hexd^act/+/+^ embryos. We reduced the levels of Nodal signalling by crossing Hexd^act/+^ and Nodal^LacZ/+^ mice [29]. In Nodal^LacZ/+^ embryos at 6.5dpc, β-galactosidase staining is posteriorly restricted and the A–P axis is correctly established (Fig.5E)[39]. In contrast to this, the majority of Hexd^act/+^; Nodal^LacZ/+^ mutants examined at 6.5dpc displayed a clear reduction in β-galactosidase staining and its expression was observed throughout the proximal epiblast (75%; n = 6/8 Fig.5E). This indicates that in these embryos there is a clear reduction in Nodal signalling activity as well as an increase in the proportion of embryos showing no posterior restriction in *Nodal* expression when compared to Hexd^act/+^ mutants (75% of Hexd^act/+^; Nodal^LacZ/+^embryos showed ectopic LacZ expression compared to 29% Hexd^act/+^ embryos that showed ectopic *Nodal* mRNA expression).

To confirm these observations we analysed *T and Fgf8* expression in Hexd^act/+^; Nodal^LacZ/+^ embryos at 7.5dpc. Hexd^act/+^ embryos on this genetic background displayed an absence of the anterior primitive streak domain of expression of *T* in 12.5% of cases analysed (n = 2/16) and of Fgf8 in 14% of cases (n = 1/7). In contrast to this 41% of Hexd^act/+^; Nodal^LacZ/+^ mutants showed a lack of *T* expression in the anterior primitive streak (n = 7/17; Fig.5F) and 54% showed a decrease in *Fgf8* expression in this domain (n = 6/11; Fig.5G). This indicates that reducing the levels of Nodal signalling increases the frequency of phenotypes observed in *Hex*-AVE-ablated embryos. It also suggests that in addition to the role of the AVE in restricting *Nodal* expression, *Hex-*AVE cells also assist in augmenting Nodal activity prior to primitive streak formation.

## Discussion

The AVE is a signalling centre required for A–P axis specification (reviewed [3], [6]). To date the AVE has been shown to have roles in inhibiting Nodal signalling, promoting forebrain identity and inhibiting primitive streak formation. The results presented here indicate that the AVE has additional roles to these ones. We find that the *Hex*-AVE is required for the correct patterning of the anterior primitive streak. Therefore the AVE, in addition to patterning the anterior of the embryo, also patterns its posterior.

The AVE has been suggested to comprise multiple cell subpopulations [4], [23]. To address the role of the *Hex*-AVE we knocked the diphtheria toxin subunit A (DTA) into the Hex locus in a Cre inducible manner. DTA catalyses the inactivation of elongation factor 2, resulting in termination of protein synthesis and cell death at very low concentrations [25], [40], [41], [42], [43]. To monitor the time of loss of the *Hex*-AVE we used the *Hex*-GFP reporter line [30], which is independent of the *Hex* locus. We found that at 5.5dpc the vast majority of embryos showed *Hex*-GFP expression but that by 6.5dpc one third of embryos had lost this expression. This indicated that in these embryos the *Hex*-AVE was ablated between 5.5dpc and 6.5dpc. When we analysed how other AVE markers were being affected by ablation of the *Hex*-AVE we observed that the expression of *Lefty1*, *Fz8* and *Cerl* were severely affected in 30% of Hexd^act/+^ embryos, suggesting that between 5.5dpc and 6.5dpc these genes are expressed in the *Hex*-AVE or that their expression is maintained by the *Hex*-AVE. In contrast to this we found no change in the expression of *Sfrp5* and *Dkk1* in Hexd^act/+^ embryos indicating that correct placement of cells expressing these genes is independent of the *Hex*-AVE after 5.5dpc. Together these observations provide strong support for the notion that the AVE is indeed composed of multiple cell subpopulations.

Our work has also shed light on what roles the AVE has on patterning the posterior of the embryo. Analysis of how ablation of the *Hex*-AVE impacted on gastrulation revealed that these cells were required to pattern the anterior primitive streak. This is indicated by the fact that 24% of Hexd^act/+^ embryos showed defects in *T*, *Eomes*, *Chordin*, *Noggin*, *Fgf8* or *Snail* expression in the anterior primitive streak between 6.5–7.5dpc, a similar proportion to the 30% of Hexd^act/+^ embryos with a miss-patterned AVE at 6.5dpc. In contrast to this, forebrain specification occurred apparently normally in all Hexd^act/+^ embryos as suggested by the normal *Otx2* expression at 7.5dpc (Fig.S3), indicating either that by 6.5dpc the *Hex*-AVE has already initiated anterior patterning in the epiblast or that other AVE subpopulations are able to perform these roles. At 9.5dpc a third of Hexd^act/+^ embryos showed mild forebrain patterning defects. These patterning defects are not as pronounced as those shown by zygotic mutations that affect genes required for AVE specification [44]. The mild forebrain defects observed in *Hex*-AVE ablated embryos could be due to either a role of the *Hex*-AVE in patterning the forebrain between 5.5 and 6.5dpc or a consequence of the subtle anterior primitive streak defects present in these embryos.

How do the primitive streak patterning defects we see in Hexd^act/+^ embryos arise? It has been shown that Wnt3 and Nodal act in an autoregulatory loop to amplify Nodal signalling in the posterior epiblast [35] and this is essential for patterning of the anterior primitive streak (reviewed [3], [6]). In this manuscript we show that in Hexd^act/+^ embryos *Nodal* and *Wnt3* expression are miss-localised, suggesting that Nodal signalling is disrupted in these embryos. We also demonstrate that reducing Nodal levels in Hexd^act/+^ embryos increases the frequency of affected embryos indicating that *Hex*-AVE ablation is causing decreased Nodal signalling. A decrease in Nodal signalling has been shown to cause anterior primitive streak defects (reviewed in [6]). Also, mutation of *Ectodermin*, an intracellular inhibitor of Nodal signalling, causes an expansion of the anterior primitive streak [45]. Together, this suggests that a decrease in Nodal signalling is the most likely cause of the primitive streak defects we observe after ablation of the *Hex*-AVE.

**Figure 6:**
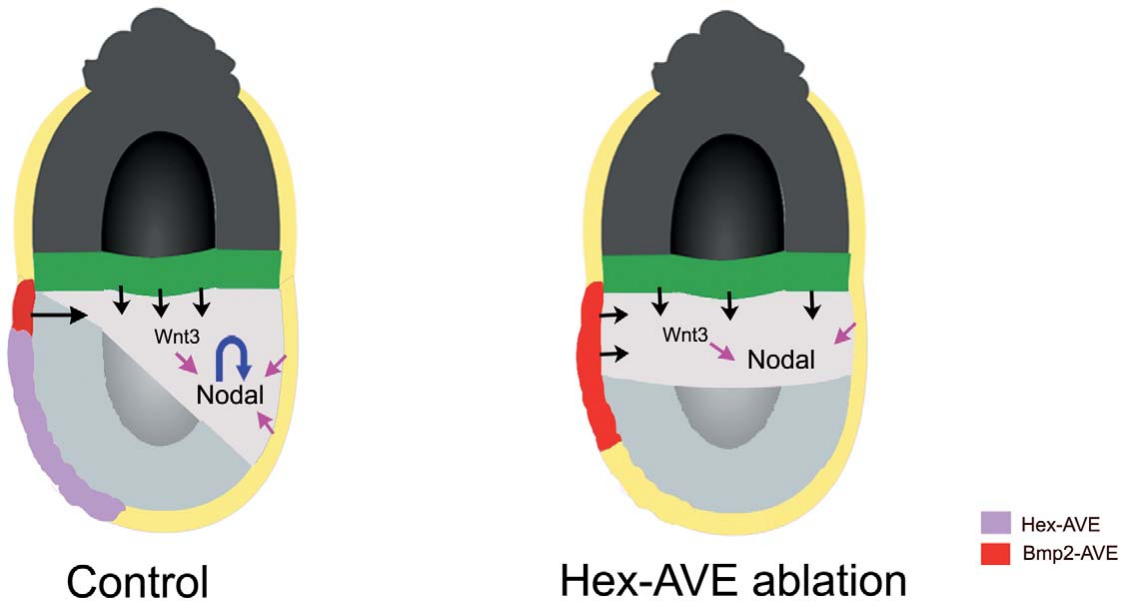
Model for how ablation of the *Hex*-AVE leads to proximal *Wnt3* and *Nodal* expression. Lack of the *Hex*-AVE leads to a failure to restrict Bmp2 expression to the proximal visceral endoderm and this leads to ectopic *Wnt3* and *Nodal* expression in the anterior epiblast. This miss-localisation of *Wnt3* and *Nodal* leads to the inability to correctly amplify *Nodal* in the posterior epiblast at 6.5dpc, causing defective specification of the anterior primitive streak.

What could be causing the miss-localisation of *Nodal* and *Wnt3* expression? We find that in Hexd^act/+^ embryos *Bmp2* expression persists in the AVE at 6.5dpc and is not restricted to the proximal boundary as occurs in control embryos. There are two alternative explanations for this observation. Either the *Hex*-AVE is required to displace those cells expressing *Bmp2* or the *Hex*-AVE is required for the downregulation of *Bmp2* expression in the AVE. Previously we, and others, have shown that BMP signalling is required to maintain *Nodal* expression in the post-implantation epiblast [35], [36]. BMP4 secreted by the extra-embryonic ectoderm has been shown to be involved in this maintainance [35]. Here we show that culturing embryos in BMP2 leads to ectopic *Nodal* expression, suggesting that BMP2 can also maintain *Nodal* expression in the epiblast. Given that the ectopic domains of *Nodal* and *Wnt3* expression in the epiblast of Hexd^act/+^ embryos are directly adjacent to the domain of the AVE where we observe ectopic *Bmp2* expression, it is tempting to speculate that the ectopic *Bmp2* expression is causing the ectopic *Nodal* expression in Hexd^act/+^ embryos (Figure 6). The failure to restrict *Nodal* expression to the posterior of the embryo is likely to lead to a lower threshold of Nodal signalling in this region of the embryo as the Nodal/Wnt3 amplification loop is disrupted, and this lower threshold of signalling will affect the specification of the anterior primitive streak derivatives that require the highest levels of signalling. In this way Nodal signalling is both upstream of the AVE, as it induces AVE gene expression [20], and downstream of this tissue, as some AVE genes, such as *Bmp2*, are required to maintain its expression. Ablation of *Bmp2* specifically in the AVE would be required to confirm this hypothesis.

In conclusion our experiments identify that the AVE is not only required for anterior patterning, but also that specific sub-populations of this tissue are required for the correct patterning of the posterior of the embryo.

## Supporting Information

Figure S1 **Marker analysis in severely affected Hexd^act/+^ embryos.** (A-B) *Hex*-GFP and *Cerl* expression is lost and (C) *Ttr* expression expanded in the visceral endoderm of severely affected Hexd^act/+^ mutant embryos. (D) At 6.5dpc *Eomes* expression is lost from the epiblast and (E) *Nodal* expression is inappropriately expressed in the anterior epiblast in severely affected Hexd^act/+^ mutant embryos. (F) *Wnt3* expression is down-regulated and restricted to the proximal epiblast and (G) *T* and (H) *Foxa2* expression is lost in severely affected Hexd^act/+^ mutant embryos at 7.5dpc. (I) At 7.5dpc *Oct4* is expressed in the epiblast of Hexd^act/+^ mutant embryos. *Cerl*, n = 1, *Ttr,* n = 2, *Eomes* n = 1, *Nodal* n = 1 *Oct4* n = 1, *Wnt3* n = 2, *T* n = 2, *Foxa2* n = 2. Scale bar 60 µm in A-C; 70 µm in D-G.
(TIF)

Figure S2 **Forebrain defects in Hexd^act/+^ embryos.** (A) No change in *Otx2* expression at 7.5dpc and (B-B′) reduced *Six3* at 8.5dpc and (C) *Foxg1* at 9.5dpc in Hexd^act^ embryos. Arrow indicates the site of forebrain patterning defects.
(TIF)

Figure S3 ***Bmp2*** **expression in wild-type and Hexd^act/+^ embryos.** (A) At 5.75dpc *Bmp2* is expressed in the AVE and posterior VE. (B-C) Different views of two Hexd^act/+^ embryos showing ectopic *Bmp2* expression at 6.5dpc.
(TIF)

Table S1 **Genotyping results of Hexd × β-actin Cre crosses at various embryonic stages and weaning age.**
(PDF)

Table S2 **Genotyping results of Hexd^act^ × +/+ crosses at various embryonic stages and weaning age.**
(PDF)

Table S3 **Proportion of Hexd^act^ embryos showing forebrain defects at 8.5dpc and 9.5dpc.**
(PDF)
