# Supplementary figures and images for: Correct Patterning of the Primitive Streak Requires the Anterior Visceral Endoderm
Source: PLoS One. 2011 Mar 18;6(3):e17620. doi: 10.1371/journal.pone.0017620 (PMC3060820; doi:10.1371/journal.pone.0017620)

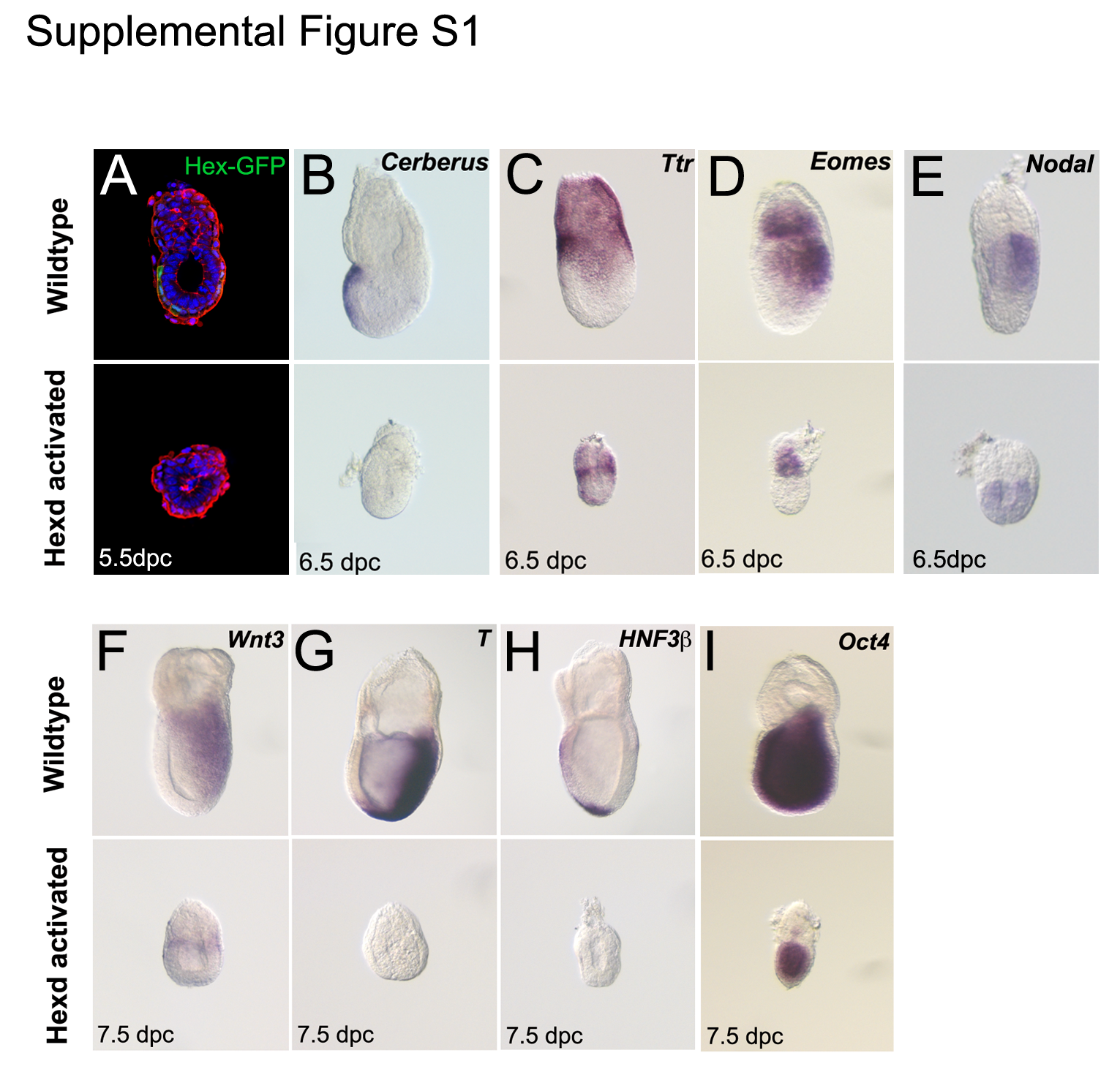

Supplement: Figure S1 — Marker analysis in severely affected Hexdact/+ embryos. (A-B) Hex-GFP and Cerl expression is lost and (C) Ttr expression expanded in the visceral endoderm of severely affected Hexdact/+ mutant embryos. (D) At 6.5dpc Eomes expression is lost from the epiblast and (E) Nodal expression is inappropriately expressed in the anterior epiblast in severely affected Hexdact/+ mutant embryos. (F) Wnt3 expression is down-regulated and restricted to the proximal epiblast and (G) T and (H) Foxa2 expression is lost in severely affected Hexdact/+ mutant embryos at 7.5dpc. (I) At 7.5dpc Oct4 is expressed in the epiblast of Hexdact/+ mutant embryos. Cerl, n = 1, Ttr, n = 2, Eomes n = 1, Nodal n = 1 Oct4 n = 1, Wnt3 n = 2, T n = 2, Foxa2 n = 2. Scale bar 60 µm in A-C; 70 µm in D-G. (TIF) [file pone.0017620.s001.tif]

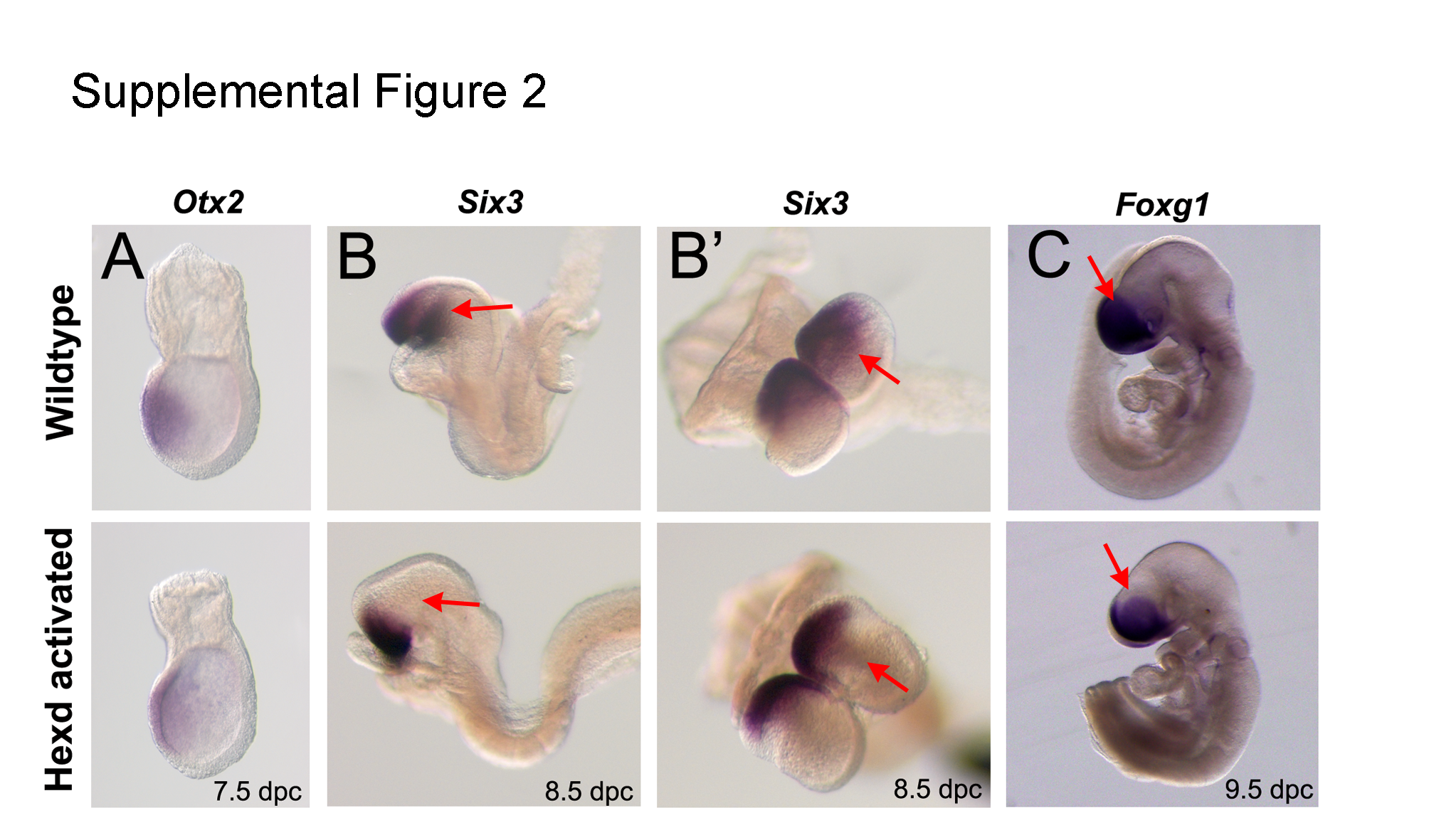

Supplement: Figure S2 — Forebrain defects in Hexdact/+ embryos. (A) No change in Otx2 expression at 7.5dpc and (B-B′) reduced Six3 at 8.5dpc and (C) Foxg1 at 9.5dpc in Hexdact embryos. Arrow indicates the site of forebrain patterning defects. (TIF) [file pone.0017620.s002.tif]

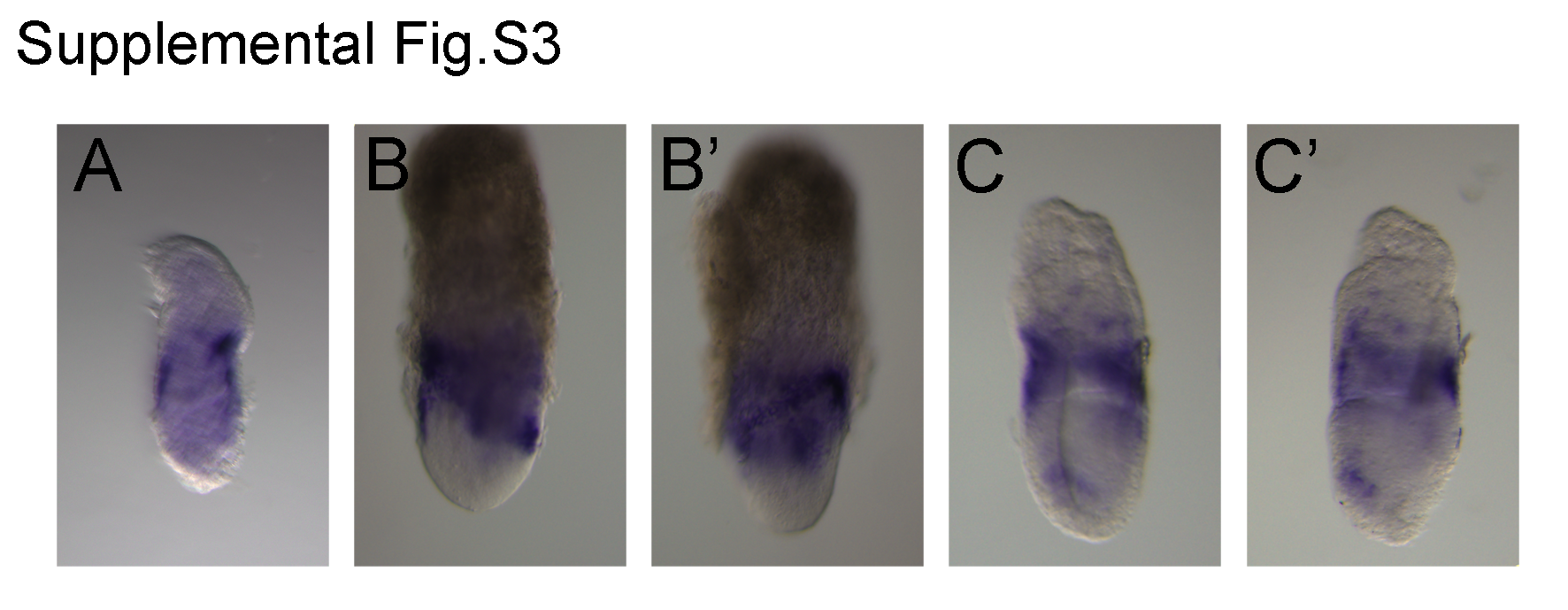

Supplement: Figure S3 — Bmp2 expression in wild-type and Hexdact/+ embryos. (A) At 5.75dpc Bmp2 is expressed in the AVE and posterior VE. (B-C) Different views of two Hexdact/+ embryos showing ectopic Bmp2 expression at 6.5dpc. (TIF) [file pone.0017620.s003.tif]
