# Supplementary material for: Correct Patterning of the Primitive Streak Requires the Anterior Visceral Endoderm
Source: PLoS One. 2011 Mar 18;6(3):e17620. doi: 10.1371/journal.pone.0017620 (PMC3060820; doi:10.1371/journal.pone.0017620)
Supplement: Table S1 — Genotyping results of Hexd × β-actin Cre crosses at various embryonic stages and weaning age. (PDF) [file pone.0017620.s004.pdf]

**Hexd × β-actin Cre**		**Genotype**	
Stage (dpc)	Total	$Hexd^{Act}$	Control
5.5	20	6 (30%)	14 (70%)
6.5	180	40 (22%)	140 (78%)
7.5	41	15 (37%)	26 (63%)
9.5	81	17 (21%)	64 (79%)
11.5	41	7 (17%)	34 (83%)
Weaning	156	18 (11%)	138 (89%)
