# Supplementary material for: Correct Patterning of the Primitive Streak Requires the Anterior Visceral Endoderm
Source: PLoS One. 2011 Mar 18;6(3):e17620. doi: 10.1371/journal.pone.0017620 (PMC3060820; doi:10.1371/journal.pone.0017620)
Supplement: Table S2 — Genotyping results of Hexdact × +/+ crosses at various embryonic stages and weaning age. (PDF) [file pone.0017620.s005.pdf]

**$Hexd^{Act}$ × +/+**		**Genotype**	
Stage (dpc)	Total	$Hexd^{Act}$	Control
Weaning	95	33 (35%)	62 (65%)
