# Supplementary material for: Correct Patterning of the Primitive Streak Requires the Anterior Visceral Endoderm
Source: PLoS One. 2011 Mar 18;6(3):e17620. doi: 10.1371/journal.pone.0017620 (PMC3060820; doi:10.1371/journal.pone.0017620)
Supplement: Table S3 — Proportion of Hexdact embryos showing forebrain defects at 8.5dpc and 9.5dpc. (PDF) [file pone.0017620.s006.pdf]

Hexd$^{Act/+}$

Stage (dpc)	Total	Forebrain defect	Normal
8.5	20	5 (25%)	15 (75%)
9.5	9	3 (33%)	6 (66%)
